# Clinical characteristics of patients with COVID-19 admitted to the COVID-19 Emergency Field Hospital of Bangkalan, Indonesia

**DOI:** 10.12688/f1000research.110716.2

**Published:** 2022-08-26

**Authors:** Erwin Astha Triyono, Fenska Seipalla, Nathania Djaja, Ahmad Maulana Ifan Akbas, Kurnia Auliyana Ar-Rahmah, Perthdyatama Syifaq Budiono, Aditya Putra Pamungkas, Yussika Fernanda, Alfin Jam'Annuri, Clarissa Azalia Maheswari

**Affiliations:** 1 COVID-19 Emergency Field Hospital of Bangkalan, Bangkalan, East Java Province, 69112, Indonesia; 2 Department of Internal Medicine, Faculty of Medicine-Dr. Soetomo Teaching Hospital Universitas Airlangga,, Surabaya, East Java Province, 60286, Indonesia

**Keywords:** COVID-19, isolation, coronavirus, pandemic, field hospital, clinical characteristics

## Abstract

**Background:** Following the surge of coronavirus disease 2019 (COVID-19) cases in the epicenter of East Java Province, this study aimed to determine the clinical characteristics of patients with COVID-19 at one of the emergency field hospitals in Indonesia.

**Methods:** This was a single-centered, retrospective descriptive study of 763 patients admitted to the COVID-19 Emergency Field Hospital of Bangkalan from July 5 2021 to September 30 2021. The demographic data, clinical signs and symptoms, pre-existing comorbidities, therapy, and clinical outcomes of the patients were analyzed using SPSS.

**Results:** The clinical characteristics of patients with COVID-19 at the emergency hospital were varied. A total of 763 patients were included. The most common age was between 40 and 49 years (31.1%), a slight majority were women (51.5%), and most had travelled abroad in the last 14 days (99.1%). Of the 763 patients, 70.9% had no comorbidities. Half of the patients were asymptomatic (49.4%), 46% were mild cases, 4.1% were moderate, and 0.5% severe. The most common symptoms were productive cough (15.7%) and headache (15.3%). Supportive and comorbidity therapy were given which showed excellent clinical outcomes.

**Conclusions:** This study presents the description of the clinical characteristics of COVID-19 patients during high surge cases of COVID-19 that are mostly dominated by Indonesian migrant workers in a field hospital. majority of COVID-19 patients were asymptomatic and therapy without antibiotics or antivirals showed positive outcomes in COVID-19 patients.

## Introduction

Coronavirus disease 2019 (COVID-19) is still a concern worldwide due to its rapid spread and enormous burden in all aspects. COVID-19 is caused by severe acute respiratory syndrome coronavirus 2 (SARS-CoV-2) ^1, 2^. On March 12, 2020, the World Health Organization (WHO) declared COVID-19 a pandemic ^3^. The pandemic has already spread to several nations throughout the world, including Indonesia. Indonesia is a middle-income country with one of the lowest per capita health expenditures in the category ^4^. Low- and middle-income countries (LMICs) are predicted to be affected most by the COVID-19 pandemic ^5^. Physical distance and lockdown are nearly impossible due to the population's poor socioeconomic standing, which is characterized by congested living circumstances, limited access to daily basic requirements (e.g., food, clean water), and reliance on daily wages. Furthermore, caring for patients with COVID-19 is difficult since already limited health-care resources are soon exhausted.

The first COVID-19 case was reported in Indonesia in 2020, until early December 2021, the total number of cases in Indonesia was approximately 4,256,687 cases with a death amounting to 143,840 people ^6^. Since March 2020, COVID-19 cases rapidly increased which made East Java, Indonesia’s second most populous province, the epicenter of the pandemic from March to June 2020, where the highest number took place in this hospital area, Bangkalan. In response, the Indonesian government established the COVID-19 Emergency Field Hospital of Bangkalan in collaboration with the East Java Provincial Government, Regional Disaster Management Agency, and Regional Public Hospital Dr. Soetomo. This hospital was the epicenter of COVID-19 in Indonesia since the highest number of cases occurred in Bangkalan. From June 2021, the Emergency Field Hospital of Bangkalan conducted a study about the characteristics of patients with COVID-19 in Indonesia since studies are still limited.

The COVID-19 Emergency Field Hospital of Bangkalan - intended to treat a number of patients with COVID-19 with asymptomatic and mild–moderate symptoms to reduce community transmission. The study regarding the clinical characteristics of patients with COVID-19 in low-resource settings such as emergency field hospitals in Indonesia was limited, and particular studies of patients with COVID-19 in the Bangkalan area had not been established to date. This report will demonstrate the clinical characteristics of patients with COVID-19 in the Bangkalan area.

Clinical manifestations of COVID-19 are varied. Some patients are asymptomatic or have mild symptoms such as fever, cough, dyspnea, myalgia, and fatigue ^7– 9^. For severe cases, there are manifestations of acute respiratory distress syndrome and a combination of other complications that lead to death ^10, 11^. The characteristics of patients in this kind of emergency hospital in LMIC settings are still largely unknown, especially during the early phase of the pandemic. This study aimed to provide information regarding the clinical characteristics of patients with COVID-19 infection who have been hospitalized during high surge of COVID-19 ina field hospital of Indonesia.

## Methodology

### Study design

This is a retrospective descriptive, non-experimental study of patients with COVID-19 admitted to the COVID-19 Emergency Field Hospital of Bangkalan, Madura, Indonesia from July 5, to September 30, 2021. This date range was selected from the opening date of service of this Hospital until the lowest COVID-19 cases rate in this hospital. All data were obtained from hospital electronic medical records, including demographic data, clinical signs and symptoms, therapy, and clinical outcomes of the patients. Only researchers of this study have access to these data to protect patient confidentiality.

### Patient criteria

Patients admitted to the COVID-19 Emergency Field Hospital of Bangkalan were diagnosed with COVID-19 infection according to the diagnostic criteria from the WHO Clinical management of COVID-19 interim guidance (27 May 2020) ^12^. All patients were tested positive using reverse-transcription polymerase chain reaction (RT-PCR) and quarantined in this hospital. The patients were then classified based on the severity of COVID-19. Data for all patients with COVID-19 in the COVID-19 Emergency Field Hospital of Bangkalan were included in this study, and there were no exclusion criteria.

### Data characteristics

All demographic data were collected from the electronic medical records, including basic information (age, sex, travel history within 14 days). Medical history including pre-existing comorbidities such as hypertension, diabetes, obesity, pregnancy, and cerebrovascular accident was recorded based on a clinical assessment or patient self-reporting in anamnesis. Clinical characteristics data including dry cough, productive cough, fever, anosmia and/or ageusia, cold, headache, nausea, vomiting, dyspnea, diarrhea, myalgia, COVID-19 RT-PCR data, treatment, and outcome were obtained from the medical records. An organized checklist was used when extracting the data from medical records to ensure complete information that was needed for this study. Microsoft Excel Version 2108 was used as a data cleaning tool in this study. A backup of original data was created before any changes. Data validation entails applying constraints to ensure the consistency of data. Any missing or invalid data was identified and analyzed during the data cleaning process. The medical record number was used to avoid duplicate data. The data was standardized to ensure that the dataset was consistent and valid.

### Data analysis

The variables analyzed into categorical variables were described as frequencies and percentages. Because patients in this study were not derived from random selection, all statistical data are deemed to be descriptive only. The data were analyzed using SPSS version 23.0 (IBM SPSS Statistics, RRID:SCR_016479) and represented in the form of tables and text.

### Ethical approval

This study received ethical approval from the Joint Ethics Committee of Dr. Soetomo Teaching Hospital and Faculty of Medicine Universitas Airlangga (Surabaya, Indonesia) with approval number 272/EC/KEPK/FKUA/2021. Written informed consent was obtained from all patients when admitted to the hospital for the usage of research purposes.

## Results

### Clinical characteristics

Table 1 presents the characteristics of 763 patients that fit the research criteria during the research period from July 5, 2021 to September 30, 2021 as found in the underlying data ^13^. Of these, 393 (51.5%) patients were female and 40–49 years (31.1%) was the most common age range (237 patients), whilst 27.1% were 30–39 years, 21.2% were 18–29 years, 16.1% were 50–64 years, 2.5% were above 64 years, and 0–17 years (2.0%) was the least common age range. As many as 756 (99.1%) patients admitted to this hospital had travelled abroad in the last 14 days, and the rest were local residents. Overall, 172 (22.5%) patients were recorded to have had at least one pre-existing comorbidity, 46 (6.0%) patients had two comorbidities, and 4 (0.5%) patients had three comorbidities. The most common comorbidity was grade 1 obesity found in 94 (12.3%) patients, followed by hypertension (11.0%), grade 2 obesity (5.9%), pregnancy (4.7%), diabetes mellitus (1.7%), and others (various comorbidities such as cerebrovascular accident, tuberculosis, and cardiovascular diseases).

**Table 1.** Demographic characteristics of patients at the COVID-19 Emergency Field Hospital of Bangkalan.

Patient demographics (n = 763)	Value (%)
Sex	
Female	393 (51.5)
Male	370 (48.5)
Age	
0–17 years	15 (2.0)
18–29 years	162 (21.2)
30–39 years	207 (27.1)
40–49 years	237 (31.1)
50–64 years	123 (16.1)
>64 years	19 (2.5)
Patient origin	
Travelers abroad	756 (99.1)
Local residents	7 (0.9)
Comorbidity	
No comorbidity	541 (70.9)
One comorbidity	172 (22.5)
Two comorbidities	46 (6.0)
Three comorbidities	4 (0.5)
Hypertension	84 (11.0)
Diabetes mellitus	13 (1.7)
Pregnancy	36 (4.7)
Grade 1 obesity	94 (12.3)
Grade 2 obesity	45 (5.9)
Others (CVA, TB, CVDs)	4 (0.5)

CVA, cerebrovascular accident; TB, tuberculosis; CVDs, cardiovascular diseases

### Signs and symptoms

Out of the 763 patients with COVID-19 treated in the COVID-19 Emergency Field Hospital of Bangkalan, 377 (49.4%) patients were asymptomatic, 351 (46%) patients had mild symptoms, 31 (4.1%) patients had moderate symptoms, and 4 (0.5%) patients had severe symptoms. Productive cough was the most frequent symptom, with a prevalence of 15.7% (120 patients), followed by headache in 117 (15.3%) patients, cold in 66 (8.7%) patients, dry cough in 45 (5.9%) patients, fever in 45 (5.9%) patients, sore throat in 36 (4.7%) patients, dyspnea in 22 (2.9%) patients, and symptoms such as diarrhea, myalgia, and abdominal pain were less common. Anosmia or ageusia, known as the most specific symptom of COVID-19 infections, was the least complained symptom, with approximately five patients or 0.7% of patients only ( Table 2). All patients included in this study were positive for SARS-CoV-2, tested using the RT-PCR method.

**Table 2.** Clinical characteristics of patients at COVID-19 Emergency Field Hospital of Bangkalan.

Characteristics (n = 763)	Value (%)
Severity	
No symptoms	377 (49.4)
Mild	351 (46)
Moderate	31 (4.1)
Severe	4 (0.5)
General symptoms	
Dry cough	45 (5.9)
Productive cough	120 (15.7)
Sore throat	36 (4.7)
Fever	45 (5.9)
Anosmia or ageusia	5 (0.7)
Cold	66 (8.7)
Headache	117 (15.3)
Nausea	17 (2.2)
Vomiting	7 (0.9)
Dyspnea	22 (2.9)
Diarrhea	12 (1.6)
Myalgia	13 (1.7)
Abdominal pain	15 (2.0)

### Therapy

The therapy given to all patients were isolation, supportive drugs (vitamin, antipyretic, antitussive, decongestant, anti-diarrhea, antiemetic), encourage patient to getting enough rest and staying well hydrated, adequate nutrition, based on Table 3. Moreover, patients with comorbidity were given therapy according to their condition. Approximately 759 (99.5%) patients received multivitamins, 163 (21.3%) patients received antipyretic therapy (such as paracetamol), 155 (20.3%) patients received cough medicine (such as N-acetylcysteine, Ambroxol, Codeine), 59 (7.7%) patients received decongestant therapy (such as Allerfed, Demacolin), and a small number of patients received anti-diarrhea (1.0%) and antiemetic therapy (0.7%). Therapy according to each patient’s comorbidity was also given in conjunction with symptomatic therapy; 112 (14.7%) patients received antihypertensive therapy, and 14 (2.8%) patients received antidiabetic therapy. Furthermore, 9 (1.3%) patients received supplemental oxygen therapy. No antiviral or corticosteroid therapy was given to any of the patients admitted to Bangkalan Emergency Field Hospital.

**Table 3.** Therapy of patients at COVID-19 Emergency Field Hospital of Bangkalan.

Characteristics (n = 763)	Value (%)
Supportive therapy	
Multivitamin	759 (99.5)
Antipyretic	163 (21.3)
Cough medicine	155 (20.3)
Decongestant	59 (7.7)
Anti-diarrhea	8 (1.0)
Antiemetic	5 (0.7)
Comorbid therapy	
Antihypertensive drug	112 (14.7)
Antidiabetic drug	14 (1.8)
O _2_ therapy	9 (1.3)

### Clinical outcomes

Out of a total of 763 patients, 692 (90.7%) patients recovered from COVID-19 with 14 days isolation from the first PCR swab, and 43 (5.6%) patients continued self-quarantine. There were 28 (3.7%) patients referred to a more equipped health facility due to desaturation (SpO _2_ <94%). No patient died during the research period. Based on the average length of stay (LOS), the largest number was in groups with less than 10 days LOS with as many as 753 (98.7%) patients, LOS for 10 days was 3 (0.4%) patients, and LOS for more than 10 days was 7 (0.9%) patients. LOS varies because based on the hospital policy, patients must be quarantined for at least ten days based on the first date of PCR test results. But some patients came with positive PCR results from external laboratories 2 or 3 days prior to their hospital admission. That explains LOS of less than ten days. Furthermore, the discharge criteria for asymptomatic patients were ten days quarantine from the first positive PCR date, and for symptomatic patients were at least ten days quarantine plus 3 additional symptom-free days. The clinical outcomes data are presented in Table 4.

**Table 4.** Clinical outcomes and length of stay of patients at COVID-19 Emergency Field Hospital of Bangkalan.

Characteristics (n = 763)	Value (%)
Clinical outcomes	
Cured	692 (90.7)
Self-quarantine	43 (5.6)
Referred	28 (3.7)
Died	0 (0)
Length of stay	
<10 days	753 (98.7)
10 days	3 (0.4)
>10 days	7 (0.9)

## Discussion

Between March and June 2020, Jakarta Special Capital Region Jakarta and East Java Province were the epicenters of COVID-19 cases in Indonesia with the highest number of cases occurring in Bangkalan ^14^. The rapid rise of COVID-19 cases might be related to the population density in East Java, which was high (851 people per km ^2^). In Bangkalan, where the highest case took place, the population density reached 1,059 people per km ^2^ ^15^. Furthermore, we found patients in adulthood (18–59 years) dominating the data reported, with 40–49 years (31.1%) being the most common age range, followed by 30–39 years (27.1%) and 18–29 years (21.2%), respectively ( Table 1). This finding was consistent with the previous study in Indonesia stating that the incidence correlates with adult people being engaged in many daily activities and actively working; therefore, it is easier for them to be infected while not strictly adhering to COVID-19 prevention protocols ^16^. This may have allowed COVID-19 to spread more greatly contributing to the sudden outbreak in East Java. In this study, the prevalence of COVID-19 was slightly higher in women (51.5%), in concordance with the East Java population where the female population was slightly higher (50.1%) and in contrast to many previous studies in which more men were infected ^15– 18^. Our study found that 99% of patients hospitalized here had a history of traveling abroad. Most of the patients who have a history of traveling abroad are Indonesian immigrant workers, in which 79.9% are women ^19^. Additionally, the overall population in East Java Province is dominated by women ^20^. This might have affected the sex dominance in our study.

Among 763 patients included in this study, 377 (49.4%) patients were asymptomatic. Moreover, 120 (15.7%) patients complained of productive cough, followed by headache in 117 (15.3%) patients, cold (8.7%), dry cough (5.9%), fever (5.9%), and sore throat (4.7%). Less common symptoms were dyspnea, nausea, abdominal pain, myalgia, diarrhea, vomiting, anosmia, and/or ageusia. These findings support the earlier studies that fever and cough were the dominant symptoms and gastrointestinal symptoms were uncommon ^18– 22^. Anosmia and/or ageusia stated to be the single strongest predictor of COVID-19 infections (by 10.2-fold higher than those with COVID-19-like illness) were found at the least number in our study. For its reliability, this symptom was used as the empiric signal for symptom-based public health surveillance in areas with fewer facilities available; however, several hypothesized that anosmia and/or ageusia mechanisms still require future studies ^23, 24^.

Overall, 172 (22.5%) patients were recorded to have at least one pre-existing comorbidity, 46 (6.0%) patients have two comorbidities, and 4 (0.5%) patients have three comorbidities. The most common comorbidity was grade 1 obesity found in 94 (12.3%) patients, followed by hypertension (11.0%), grade 2 obesity (5.9%), pregnancy (4.7%), diabetes mellitus (1.7%), and others. Previous studies also mentioned risk factors contributing to more severe disease and poorer outcomes with COVID-19 infections such as age >60 years, obesity, hypertension, diabetes, cardiovascular diseases, cerebrovascular diseases, chronic kidney disease, and chronic obstructive pulmonary disease ^25, 26^. This study also collected data on patients’ comorbidities, with obesity becoming the most common (12.3%). Evidence from a previous study involving 4,265 patients with COVID-19 in Jakarta stated that the risk of death across all ages was higher for patients with more than one comorbidity than for those without; the risk among patients <50 years was notably increased by six-fold ^25^. In addition, pregnancy is widely considered as a comorbidity; the most common comorbidity in this study was obesity. It correlates with this study finding since a large number of patients present with fewer comorbidities, mostly asymptomatic, and therefore come with great clinical outcomes.

When this study was conducted, several drugs had been repurposed for COVID-19 treatment, yet no effective treatment against the virus had been proven ^23^. Supportive therapy and monitoring remain the mainstay treatment of mild and asymptomatic COVID-19 infections. Suspected or confirmed mild COVID-19 should be isolated, given treatment such as antipyretics for fever and pain, adequate nutrition, appropriate rehydration, without antibiotic therapy or prophylaxis based on the latest WHO clinical management living guidance ^27^. The clinical outcomes of this study showed that 692 (90.1%) patients recovered, 43 (5.6%) patients self-quarantined, 28 (3.7%) patients referred to a more equipped health facility, and no patients died. No patient admitted to this hospital received antiviral, antibiotics, or corticosteroids. A study involving 500 patients observed disadvantages of the use of antiviral drugs for patients with COVID-19 with mild symptoms. The study concluded that antiviral treatment did not provide superior clinical outcomes to supportive care, since patients with mild COVID-19 who had received antiviral medication had significant LOS at the hospital compared with those without ^28^. Effective COVID-19 drugs were not available at the moment during our research time. Approximately 1.3% of patients receive O _2_ therapy during their stay in the hospital. This is differs with a study in Japan where 61.6% of patients did not receive respiratory support during hospitalization ^29^. The clinical outcomes were excellent; 90.1% of patients recovered.

In the current study, there were 753 (98.7%) patients with an LOS of less than 10 days, 3 (0.4%) patients with an LOS of 10 days, and 7 (0.9%) patients with an LOS of more than 10 days. Although the clinical symptoms of patients are similar to the data reported from previous studies ^17, 18^, the mortality rates reported were significantly different (no deaths reported in this study) as most study subjects had either mild or asymptomatic COVID-19 infection, and those with moderate to severe infection were referred to a more equipped health facility.

This study had some limitations. First, recall bias was inevitable based on the nature of the retrospective study. Second, the data were collected from the electronic health record database. This precluded the level of detail possible with a manual medical record review. Third, the subjects either were asymptomatic or had mild symptoms and there was no comparison to patients who had been treated with antiviral therapy. Finally, this was a small study that only covers one area where the majority of patients are Madurese and Javanese, so it falls short to represent the whole country. A further multi-center study covering more areas and patients will give a more comprehensive finding of the characteristics as well as the management of COVID-19 in Indonesia.

## Conclusion

Most patients with COVID-19 were asymptomatic with an age range between 40 and 49 years, dominated by women. Most patients had a history of travel abroad and had no comorbidities. Supportive therapy without antibiotics or antivirals was given to patients with an LOS of fewer than 10 days, and most of the cases showed excellent clinical outcomes. Findings from the data reported that our population originated from a single geographic area and may not reflect risk factors associated with clinical outcomes associated with SARS-CoV-2 infection in the general population. Accordingly, our data should be interpreted with caution as the risk factors associated with these health outcomes may differ elsewhere. Our study is a retrospective study based on electronic medical records collected during high surge cases of COVID-19 that are mostly dominated by Indonesian migrant workers. More clinical and basic research for the assessment, risk factors, and treatment of patients with COVID-19 with a wider population is needed in the future.

## Data availability

### Underlying data

Figshare: Data Clinical Characteristics of Patients with COVID-19 Admitted to the COVID-19 Emergency Field Hospital of Bangkalan, Indonesia.xlsx. https://doi.org/10.6084/m9.figshare.19337915 ^13^.

Data are available under the terms of the Creative Commons Attribution 4.0 International license (CC-BY 4.0).
