# Supplementary material for: Clinical characteristics of patients with COVID-19 admitted to the COVID-19 Emergency Field Hospital of Bangkalan, Indonesia
Source: F1000Res. 2022 Aug 26;11:414. Originally published 2022 Apr 12. [Version 2] doi: 10.12688/f1000research.110716.2 (PMC9490276; doi:10.12688/f1000research.110716.2)
Supplement: Peer review history

# Peer review: Reviewer response for version 2

**DOI:** 10.5256/f1000research.137400.r148642

**Published:** 2022-09-20

**Authors:** Petrick Periyasamy

**Affiliations:** 1 Medical Department, Faculty of Medicine, Universiti Kebangsaan Malaysia, Kuala Lumpur, Malaysia

It is good for indexing.

Is the work clearly and accurately presented and does it cite the current literature?

Partly

If applicable, is the statistical analysis and its interpretation appropriate?

Partly

Are all the source data underlying the results available to ensure full reproducibility?

Yes

Is the study design appropriate and is the work technically sound?

Partly

Are the conclusions drawn adequately supported by the results?

Partly

Are sufficient details of methods and analysis provided to allow replication by others?

Yes

Reviewer Expertise:

NA

I confirm that I have read this submission and believe that I have an appropriate level of expertise to confirm that it is of an acceptable scientific standard.

# Peer review: Reviewer response for version 2

**DOI:** 10.5256/f1000research.137400.r148643

**Published:** 2022-08-26

**Authors:** Zhongheng Zhang

**Affiliations:** 1 Department of Emergency Medicine, Sir Run Run Shaw Hospital, Zhejiang University School of Medicine, Hangzhou, China

I think the authors have made sufficient revision to my previous comments. The current version is significantly improved.

Is the work clearly and accurately presented and does it cite the current literature?

Yes

If applicable, is the statistical analysis and its interpretation appropriate?

Yes

Are all the source data underlying the results available to ensure full reproducibility?

Partly

Is the study design appropriate and is the work technically sound?

Yes

Are the conclusions drawn adequately supported by the results?

Yes

Are sufficient details of methods and analysis provided to allow replication by others?

Yes

Reviewer Expertise:

Emergency and critical care

I confirm that I have read this submission and believe that I have an appropriate level of expertise to confirm that it is of an acceptable scientific standard.

# Peer review: Reviewer response for version 1

**DOI:** 10.5256/f1000research.122349.r141325

**Published:** 2022-07-12

**Authors:** Zhongheng Zhang

**Affiliations:** 1 Department of Emergency Medicine, Sir Run Run Shaw Hospital, Zhejiang University School of Medicine, Hangzhou, China

The study is a descriptive analysis of clinical characteristics of COVID-19 patients in a single center. The methodology is sound and valid; however, much of the literature is missing because there have been several similar such studies in the literature. The study design is simple and valid; it is generally descriptive and gives important information on the clinical characteristics of COVID-19 in a special hospital. However, it is not known whether the current findings are extrapolated to other centers and regions. The methodology and analysis are simple in its current form. All statistical analyses are descriptive. It gives general description of the clinical characteristics of these patients. The conclusion is supported by the data analysis. The conclusion that therapy without antibiotics or antivirals showed positive outcomes in COVID-19 patients, is useful for clinical practice. While the study is technically sound, it lacks novelty because there has been large number of study descriptive studies in the literature.

**Some detailed comments are as follows:**

1. The conclusion in the abstract is not well formulated, it appears like a result.
2. "The COVID-19 Emergency Field Hospital of Bangkalan has a total of 242 beds and can accommodate up to a maximum of 500 beds."
  I do not think this can be described in the introduction section.
3. "This report will demonstrate the clinical characteristics needed to prevent further transmission of the virus in the Bangkalan area."
  This study purpose is not fulfilled because there is no effectiveness inference.
4. "The results of this study are expected to aid in risk stratification of patients with COVID-19 infection"
  There has been numerous risk stratification studies for COVID-19. You need to highlight how this study adds to the existing literature. The authors failed to do so.
5. "Statistical analysis"
  This section only provides descriptive analysis. Since one purpose of your study is risk stratification, you need to build a prediction model for this.

Is the work clearly and accurately presented and does it cite the current literature?

Yes

If applicable, is the statistical analysis and its interpretation appropriate?

Yes

Are all the source data underlying the results available to ensure full reproducibility?

Partly

Is the study design appropriate and is the work technically sound?

Yes

Are the conclusions drawn adequately supported by the results?

Yes

Are sufficient details of methods and analysis provided to allow replication by others?

Yes

Reviewer Expertise:

Emergency and critical care

I confirm that I have read this submission and believe that I have an appropriate level of expertise to confirm that it is of an acceptable scientific standard.

# Response

**Published:** 2022-08-11

**Authors:** Fenska Seipalla

**Affiliations:** Airlangga University, Indonesia

*Thank you for taking the time to review our paper. We appreciate your feedback very much and are grateful for the insightful comments on and valuable improvements to our paper. Below are our responses to your feedback.*

1. *"The conclusion in the abstract is not well formulated, it appears like a result."* 
  > Thank you for the feedback. We will revise that part.
2. *""The COVID-19 Emergency Field Hospital of Bangkalan has a total of 242 beds and can accommodate up to a maximum of 500 beds."*
*I do not think this can be described in the introduction section." *
  > Thank you for the feedback. We will revise the introduction section.
3. *""This report will demonstrate the clinical characteristics needed to prevent further transmission of the virus in the Bangkalan area." This study purpose is not fulfilled because there is no effectiveness inference."*
  > Yes, we agree with your suggestion. We will revise it. Thank you.
4. *""The results of this study are expected to aid in risk stratification of patients with COVID-19 infection"*
* There has been numerous risk stratification studies for COVID-19. You need to highlight how this study adds to the existing literature. The authors failed to do so."* 
  > Yes, we agree with your suggestion. We will revise it. Thank you.
5. *""Statistical analysis"*
* This section only provides descriptive analysis. Since one purpose of your study is risk stratification, you need to build a prediction model for this."*
  > Yes, we agree with your suggestion. We will revise the word. Thank you.

# Peer review: Reviewer response for version 1

**DOI:** 10.5256/f1000research.122349.r136850

**Published:** 2022-05-10

**Authors:** Petrick Periyasamy

**Affiliations:** 1 Medical Department, Faculty of Medicine, Universiti Kebangsaan Malaysia, Kuala Lumpur, Malaysia

This paper is relevant as it shows the real situation of clinical signs and symptoms of a middle-income country in a setting of an Emergency Field Hospital. The highlight of this paper is the number of asymptomatic patients and the paucity of symptoms. This has considerable value in terms how contact tracing needs to be reevaluated moving forward in this group for future outbreaks in this region.

 However, some clarifications are needed:

1. A death rate of 143,840 people - I think the word 'rate' may not be suitable as there is no rate here, I suggest death 'amounting to'.
2. This report will demonstrate the clinical characteristics needed to prevent further transmission of the virus in the Bangkalan area. I think this part was not mentioned in the conclusion. Would suggest that based on the findings above, there is no clinical characteristics needed to prevent further transmission of the virus?
3. Table 2 – needs some clarification – how was it broken down for example – fever only 5.9%? Out of what total? Out of the symptomatic group? How was table 2 tabulated?
4. Therapy – relaxation? What is the relaxation? I suggest you omit.
5. 43 (5.6%) patients continued self-quarantine - why was this so? It is not clear to me the intention of this self-quarantine and why?
6. Largest number was in groups with less than 10 days LOS with as many as 753 (98.7%) patients, LOS for 10 days was 3 (0.4%) patients, and LOS for more than 10 days was 7 (0.9%) patients - it is not clear to me why some were less than 10 days and some stayed longer than 10 days? What is the reason? What is the discharge criteria – that should be stated earlier?
7. Mostly asymptomatic, and therefore come with great clinical outcomes - I suggest changing this to 'and therefore had good clinical outcomes'.
8. There is no proven effective treatment against the virus to date approved by WHO - I think the timing of this sentence is misplaced as we all know there are fantastic medicines for this in 2022… so suggest rewording to mention that during that time drugs were not available?
9. A study involving 500 patients observed disadvantages of the use of antiviral drugs for patients with COVID-19 with mild symptoms. The study concluded that antiviral treatment did not provide superior clinical outcomes to supportive care, since patients with mild COVID-19 who had received antiviral medication had significant LOS at the hospital compared with those without ^^- again I would not use this reference with what we know with Paxlovid etc. in the year 2022.
10. Approximately 1.3% of patients receive O _2_ therapy during their stay in the hospital. This is consistent with a study in Japan where 61.6% of patients did not receive respiratory support during hospitalization. The clinical outcomes were excellent; 90.1% of patients recovered. The word 'consistent' may be too strong here considering less than 2% in this paper cohort progressed but 40% in the Japanese cohort progressed. I would use the word 'differs' instead.
11. Moderate to severe infection were referred to in a more equipped health facility - it would be nice to know what happened to this group if it’s possible.

Is the work clearly and accurately presented and does it cite the current literature?

Partly

If applicable, is the statistical analysis and its interpretation appropriate?

Partly

Are all the source data underlying the results available to ensure full reproducibility?

Yes

Is the study design appropriate and is the work technically sound?

Partly

Are the conclusions drawn adequately supported by the results?

Partly

Are sufficient details of methods and analysis provided to allow replication by others?

Yes

Reviewer Expertise:

infectious diseases

I confirm that I have read this submission and believe that I have an appropriate level of expertise to confirm that it is of an acceptable scientific standard, however I have significant reservations, as outlined above.

# Response

**Published:** 2022-05-13

**Authors:** Fenska Seipalla

**Affiliations:** Airlangga University, Indonesia

Thank you for taking the time to review our paper. We appreciate your feedback very much and are grateful for the insightful comments on and valuable improvements to our paper. Below are our responses to your feedback.

1. A death rate of 143,840 people - I think the word 'rate' may not be suitable as there is no rate here, I suggest death 'amounting to'.-> *We will revise the word “rate” to “amounting to.” Thank you for your feedback.*
2. This report will demonstrate the clinical characteristics needed to prevent further transmission of the virus in the Bangkalan area. I think this part was not mentioned in the conclusion. Would suggest that based on the findings above, there is no clinical characteristics needed to prevent further transmission of the virus?-> *Yes, you are correct. Thank you for the suggestion.*
3. Table 2 – needs some clarification – how was it broken down for example – fever only 5.9%? Out of what total? Out of the symptomatic group? How was table 2 tabulated? -> *Table 2 shows the total COVID-19 cases, including asymptomatic and symptomatic. The general symptoms were calculated out of all COVID-19 cases, which include asymptomatic and symptomatic cases (763 samples).*
4. Therapy – relaxation? What is the relaxation? I suggest you omit.-> *In this context, the word “relaxation” refers to getting enough rest and staying well hydrated. We will revise the word. Thank you for your suggestion.*
5. 43 (5.6%) patients continued self-quarantine - why was this so? It is not clear to me the intention of this self-quarantine and why?-> *We advise patients to extend their isolation at home (self-quarantine) for those who have already finished ten days of quarantine in hospital with no symptoms but still have second positive PCR test results for COVID-19.*
6. Largest number was in groups with less than 10 days LOS with as many as 753 (98.7%) patients, LOS for 10 days was 3 (0.4%) patients, and LOS for more than 10 days was 7 (0.9%) patients - it is not clear to me why some were less than 10 days and some stayed longer than 10 days? What is the reason? What is the discharge criteria – that should be stated earlier?-> *Our hospital policy was to quarantine patients at least for ten days based on the first date of PCR test results. Sometimes, we received patients who already had positive PCR test results from the external lab for 2 or 3 days before going to the hospital. That was why there were LOS of less than ten days. The discharge criteria were ten days quarantine for the asymptomatic patient (the first day was counted based on the first PCR date) and at least ten days quarantine + 3 additional days without symptoms for symptomatic patients.*
7. Mostly asymptomatic, and therefore come with great clinical outcomes - I suggest changing this to 'and therefore had good clinical outcomes'.-> *Yes, we agree with your suggestion. We will revise the word. Thank you.*
8. There is no proven effective treatment against the virus to date approved by WHO - I think the timing of this sentence is misplaced as we all know there are fantastic medicines for this in 2022… so suggest rewording to mention that during that time drugs were not available?-> *Yes, we agree with your suggestion. We will revise it. Thank you.*
9. A study involving 500 patients observed disadvantages of the use of antiviral drugs for patients with COVID-19 with mild symptoms. The study concluded that antiviral treatment did not provide superior clinical outcomes to supportive care, since patients with mild COVID-19 who had received antiviral medication had significant LOS at the hospital compared with those without ^^- again I would not use this reference with what we know with Paxlovid etc. in the year 2022.-> *Yes, we agree with your suggestion. We will revise it. Thank you.*
10. Approximately 1.3% of patients receive O _2_ therapy during their stay in the hospital. This is consistent with a study in Japan where 61.6% of patients did not receive respiratory support during hospitalization. The clinical outcomes were excellent; 90.1% of patients recovered. The word 'consistent' may be too strong here considering less than 2% in this paper cohort progressed, but 40% in the Japanese cohort progressed. I would use the word 'differs' instead.-> *Yes, We will revise the word. Thank you for your suggestion.*
11. Moderate to severe infection were referred to in a more equipped health facility - it would be nice to know what happened to this group if it’s possible.-> *Unfortunately, we did not have a follow-up record after referring those patients to a more equipped health facility.*
